# ^18^F-FDG positron emission tomography/computed tomography of cardiac implantable electronic device infections

**DOI:** 10.1007/s12350-020-02256-4

**Published:** 2020-07-31

**Authors:** Soile Pauliina Salomäki, Antti Saraste, Jukka Kemppainen, Saija Hurme, Juhani Knuuti, Pirjo Nuutila, Marko Seppänen, Anne Roivainen, Juhani Airaksinen, Tiina Salo, Jarmo Oksi, Laura Pirilä, Ulla Hohenthal

**Affiliations:** 1 Division of Medicine, Turku University Hospital, P.O. Box 52, 20521 Turku, Finland; 2 Turku PET Centre, University of Turku, Turku, Finland; 3 Turku PET Centre, Turku University Hospital, Turku, Finland; 4 Heart Centre, Turku University Hospital, Turku, Finland; 5 Department of Clinical Medicine, Faculty of Medicine, University of Turku, Turku, Finland; 6 Department of Physiology and Nuclear Medicine, Turku University HospitalTurku University Hospital, Turku, Finland; 7 Department of Biostatistics, University of Turku, Turku, Finland

**Keywords:** Infection, PET, molecular imaging, diagnostic and prognostic application, image interpretation

## Abstract

**Background:**

The diagnosis of cardiac implantable electronic device (CIED) infection is challenging because of its variable presentations. We studied the value of 2-[^18^F]fluoro-2-deoxy-*D*-glucose (^18^F-FDG) positron emission tomography/computed tomography (PET/CT) in the detection of CIED infection.

**Methods and results:**

Thirty patients with suspected CIED infection underwent ^18^F-FDG-PET/CT. The control group was ten patients with asymptomatic CIED who underwent cancer-related ^18^F-FDG-PET/CT. ^18^F-FDG-PET/CT was evaluated visually, semiquantitatively as maximum standardized uptake value (SUV_max_) and target-to-background ratio (TBR). Final diagnosis of CIED infection was based on clinical and bacteriological data. ^18^F-FDG-PET/CT was visually positive in all 9 patients with recent (≤ 8 weeks) implantation of CIED, but only 4 had confirmed CIED infection. ^18^F-FDG-PET/CT was true positive in 9 out of 21 cases with remote implantation of CIED and false positive in 3 (14.3%) cases. ^18^F-FDG-PET/CT was also false positive in 3 (30%) cases of control group. The SUV_max_ of the pocket area was significantly higher in patients with CIED infection than in the control group (4.8 ± 2.4 vs 2.0 ± .8, *P* < .001). By using the cut-off value of TBR ≥ 1.8, sensitivity of ^18^F-FDG-PET/CT for the diagnosis of CIED infection in patients with remote implantation was 90% and specificity 73%, PPV 75%, and NPV 89%.

**Conclusions:**

^18^F-FDG-PET/CT is a sensitive but nonspecific method in the diagnosis of CIED infection.

**Electronic supplementary material:**

The online version of this article (10.1007/s12350-020-02256-4) contains supplementary material, which is available to authorized users.

## Introduction

Clinical symptoms and manifestation of cardiac implantable electronic device (CIED) infections vary widely depending on causative microorganisms, time from implantation and patient-related factors. Symptoms can be mild and unspecific leading to delayed diagnosis associated with increasing risk of complications. Lead associated infective endocarditis occurs in less than 10% of CIED infections.^1^,^2^ In more than one third of CIED infections the pocket site can appear intact.^2^ Furthermore, it can be challenging to differentiate superficial wound infection from deep infection of the whole CIED system. Extraction of the CIED system is the recommended therapy for definitive CIED infection in most cases. Nonetheless, extraction of the whole CIED system is associated with a mortality rate of .8% and 1.5-2% risk of major complications.^3^,^4^ Thus, accurate diagnosis of CIED infection is important for timely therapy.

Multimodality imaging may help in the detection of prosthetic valve infective endocarditis, but its role in CIED infection is still uncertain.^5^ Positron emission tomography/computed tomography (PET/CT) with glucose analogue 2-[^18^F]fluoro-2-deoxy-*D*-glucose (^18^F-FDG) has been increasingly used to detect inflammation and infection. ^18^F-FDG accumulation at infection site is based on high glucose uptake of activated inflammatory cells. Recent studies have indicated potential role for ^18^F-FDG-PET/CT in diagnosis of CIED infections.^6^-^11^ We wanted to further study the diagnostic value of ^18^F-FDG-PET/CT imaging of suspected CIED infection. We evaluated patients with local signs of CIED infection after recent (≤ 8 weeks) or remote (>8 weeks) implantation of the device as well as patients presenting with fever of unknown origin, but with no local signs of CIED infection.

## Methods

### Patients

This prospective study evaluated 30 patients admitted to Turku University Hospital, Turku, Finland, between March 2011 and December 2014 due to suspected CIED infection. Patients were included consecutively with the exception of disruptions due to holidays and interruptions in the operation of FDG-PET imaging. Patients with hemodynamic instability or need for urgent surgery or extraction of the CIED were excluded. Ten patients without signs or symptoms of CIED infection who underwent ^18^F-FDG-PET/CT as part of cancer investigation between March 2011 and December 2014 were studied as controls. The hospital is a tertiary-care center for treatment of endocarditis. The study was approved by the institutional ethical review board, and all participants signed an informed consent. The study was registered as a clinical trial NCT01878721.

Clinical data were collected from all patients including history of CIED implantation and later interventions, patients’ symptoms and signs on admission, and time to PET/CT from the onset of antimicrobial treatment (Table 1). We also gathered data on microbiological findings from blood cultures, pocket wound/pus bacterial cultures and microbiological data from samples taken from CIED leads or pocket in case the device was extracted. Transthoracic echocardiography (TTE) or transesophageal echocardiography (TEE) was done to most of the patients (Table 2). Follow-up information after ^18^F-FDG-PET/CT was collected until December 2017.

**Table 1:** Characteristics of the study population and control patients

	Study population, N = 30	Control patients, N = 10
Male	23 (77)	7 (70)
Age	70 ± 13	73 ± 3
Atrial fibrillation	15 (50)	5 (50)
Coronary artery disease	6 (20)	3 (30)
Congestive heart failure	7 (23)	2 (20)
Prosthetic valve	2 (7)	0 (0)
Diabetes mellitus	12 (40)	2 (20)
Chronic renal failure	5 (17)	1 (10)
Smoking	5 (17)	2 (20)
Immunosuppressive medication	2 (7)	0 (0)
Warfarin	13 (43)	3 (30)
Aspirin (100 mg/day)	6 (20)	2 (20)
Low-molecular heparin/novel oral anticoagulant	4 (13)	1 (10)
*Type of device*		
Permanent pacemaker	23 (77)	9 (90)
Implantable cardioverter defibrillator	4 (13)	0
Cardiac resynchronization therapy-defibrillator	3 (10)	1 (10)
Two or more leads	20 (67)	6 (60)
*Last intervention before presentation*		
Implantation	18 (60)	7 (70)
Changing generator	7 (23)	3 (30)
Adding lead with or without changing generator	5 (17)	0

Values are *N* (%) or mean ±SD

**Table 2:** ^18^F-FDG-PET/CT results bacterial findings of study population and control patients

Patient no	Time from last intervention	Blood culture	Device extracted	Bacterial culture from pocket	Bacterial culture from CIED system	TEE/TTE -clinical findings	PET/CT pocket visual analysis (SUV_m_/TBR)	PET/CT Lead visual analysis (SUV_m_/TBR)	FD
*Group 1. Local* ± *general signs of infection ≤ 8 weeks after operation*									
Local signs of infection									
1	5 w	Neg	No	–	–	ND	Pos (3.5/1.3)	Pos (3.9/1.4)	FP
2	6 w	ND	No	–	–	ND	Pos (5.4/3.2)	Neg (2.4/1.4)	FP
3	2 w	Neg	Yes	*S. aureus*	Neg	TEE neg	Pos (5.7/1.8)	Pos (5.0/1.6)	TP
4	3 w	Neg	No	–	–	ND	Pos (4.8/1.5)	Pos (3.9/1.2)	FP
5	4 w	Neg	No	–	–	ND	Pos (6.0/2.2)	Neg (2.2/.8)	FP
6	3 w	Neg	No	–	–	ND	Neg (2.5/.7)	Pos (3.2/.9)	FP
Local + general signs of infection									
7	1 w	*S. aureus*	Yes	*S. aureus*	Neg	TTE neg	Pos (8.0/3.6)	Pos (7.6/3.5)	TP
8	3 w	Neg	Yes	*S. aureus*	ND	TTE neg	Pos (6.5/2.3)	Pos (3.7/1.3)	TP
9	4 w	*S. aureus*	Yes	ND	ND	TEE neg	Pos (6.3/3.3)	Pos (3.3/1.7)	TP
*Group 2. Local signs of infection/vegetation in TEE and fever > 8 weeks after operation*									
10	8 y	Neg	Yes	Neg	*S. epidermidis*	TTE neg	Pos (7.1/3.2)	Neg (2.0/.9)	TP
11	5 y	Neg	Yes	Neg	*S. epidermidis*	TEE neg	Pos (4.9/1.9)	Pos (3.5/1.3)	TP
12	2 y	ND	Yes	Neg	Neg	TEE neg	Pos (8.4/3.2)	Pos (11.5/4.4)	TP
13	2 y	Neg	Yes	Neg	*S. epidermidis*	TEE neg	Neg (2.3/1.4)	Pos (5.1/3.2)	TP
14	1.5 y	Neg	Yes	*S. aureus*	–	TEE neg	Pos (5.3/1.7)	Neg (3.0/1.0)	TP
15	5 y	Neg	Yes	ND	Neg	–	Pos (5.7/2.0)	Neg (2.8/1.0)	TP
16	6 y	Neg	Yes	Neg	Neg	TEE neg	Pos (7.5/3.8)	Neg (2.3/1.2)	TP
17	7 y	*Serratia marcescens*	No	–	–	TEE, lead thrombus	Neg (2.3/1.5)	Neg (1.5/1.0)	TN
18	5 y	Neg	Yes	ND	*Corynebacterium*	TEE, lead vegetation	Pos (2.5/1.2)	Pos (6.9/3.3)	TP
*Group 3. Fever of unknown origin or bacteremia without identified focus*									
Fever of unknown origin									
19	3 m	Neg	No	–	–	TTE neg	Neg (2.4/.7)	Neg (3.5/1.0)	TN
20	2 m	Neg	No	–	–	TEE. neg	Neg (1.6/.8)	Neg (2.8/1.3)	TN
21	2 m	Neg	No	–	–	TEE neg	Neg (2.4/1.0)	Neg (1.7/.7)	TN
22	6 m	Neg	Yes	Neg	*S. epidermidis*	TEE neg	Neg (2.6/.8)	Neg (3.2/1.0)	FN
23	15 m	Neg	Yes	ND	*S. epidermidis*	TEE neg	Neg (1.9/.8)	Pos (5.2/2.3)	TP
24	3 m	Neg	No	–	–	TEE neg	Neg (1.4/.7)	Pos (3.2/1.5)	FP
Bacteremia									
25	4 y	*S. aureus*	No	–	–	TEE neg	Neg (2.1/.8)	Pos (5.4/2.0)	FP
26	3 y	*S. aureus*	No	–	–	TEE neg	Neg (2.4/1.0)	Pos (3.8/1.5)	FP
27	5 y	*S. infantarius*	No	–	–	TEE neg	Neg (2.3/.7)	Neg (2.4/.8)	TN
28	1 y	*S. dysgalactiae*	No	–	–	TEE neg	Neg (2.0/.9)	Neg (2.4/1.1)	TN
29	8 y	*S. aureus*	No	–	–	TEE neg	Neg (2.3/.9)	Neg (1.7/.7)	TN
30	5 y	*S. aureus*	No	–	–	TTE neg	Neg (1.2/.5)	Neg (2.6/1.0)	TN
*Control patients*									
C1	2 y						Neg (1.3/.54)	Neg (2.4/1.0)	TN
C2	2.5 y						Neg (1.6/.84)	Neg (1.6/.84)	TN
C3	1.5 y						Neg (1.2/.54)	Neg (2.4/1.1)	TN
C4	.8 y						Neg (2.2/.88)	Pos (3.5/1.4)^a^	FP
C5	8 y						Neg (1.8/.78)	Neg (2.5/1.1)	TN
C6	5 y						Neg (1.6/.70)	Neg (2.0/.87)	TN
C7	6 y						Neg (1.6/.64)	Neg (2.3/.92)	TN
C8	.5 y						Pos (3.4/1.4)^b^	Neg (2.7/1.1)	FP
C9	4 y						Pos (3.5/1.8)^b^	Neg (2.0/1.1)	FP
C10	1.5 y						Neg (2.0/.8)	Neg (2.5/1.0)	TN

*w*, weeks, *m*, months, *y*, years, *ND* not done, *S. epidermidis Staphylococcus epidermidis*, *S. aureus Staphylococcus aureus*, *TEE* transesophageal echocardiography, *TTE* transthoracic echocardiography, *FD* final assessment (PET/CT results compared to final clinical diagnosis), *TP* true pos, *TN* true neg, *FN* false neg, *FP* false pos

^a^Mild uptake of ^18^F-FDG in the stem of the lead

^b^Mild uptake of ^18^F-FDG in subcutaneously/skin, no signs of infection clinically

We divided patients into three groups according to clinical presentation (Table 2). The first group (Group 1.) included patients with suspected CIED infection within 8 weeks after device implantation. The second group (Group 2.) included CIED patients with local signs of infection or with possible endocarditis more than 8 weeks after implantation. CIED related infective endocarditis was defined according to modified DUKE criteria.^5^,^12^ In the third group (Group 3) there were patients without local signs of CIED infection and either fever of unknown origin (FUO), recurrent bacteremia or bacteremia of pathogen which is typical cause of endocardial infection.

### ^18^F-FDG-PET/CT

A whole-body ^18^F-FDG-PET/CT scan (Discovery VCT, General Electric Medical Systems, Milwaukee, WI, USA) was performed in all patients. Patients with suspected CIED infection were on low-carbohydrate diet for 24 hours before the PET/CT and fasted at least 10 hours before the study to reduce physiological glucose uptake of the myocardium.^13^,^14^ Mean injected dose of ^18^F-FDG was 304 MBq (range 209-405 MBq, ±58MBq) in study group. An average of 62 minutes (range 45-100 minutes ±13 minutes) later, a whole-body PET acquisition (3 minutes per bed position) was performed following CT scan for anatomical reference and attenuation correction. In the control group there was not any specific diet before PET/CT, but procedure was otherwise the same. In control group mean injected dose was ^18^F-FDG 318 MBq (range 199-416 MBq, ± 78 MBq) and PET/CT started mean 55 minutes (range 49-76 minutes, ± 8 minutes) later. Blood glucose levels were < 10 mmol/L prior to injection of the tracer in all patients. PET images were reconstructed with 128 × 128 matrix size in full 3D mode using maximum-likelihood reconstruction with ordered-subsets expectation maximization algorithm (VUE Point, GE Healthcare).

Visual analysis of the images was performed by an experienced nuclear medicine specialist and results were re-evaluated by the research team for consensus in both populations. A positive finding was defined as a local increase in ^18^F-FDG accumulation in the CIED pocket area or in the lead. The presence of visual FDG uptake in attenuation corrected images was confirmed in non-attenuation corrected images when appropriate. In addition to the CIED system, the images were evaluated for the presence of abnormal ^18^F-FDG accumulation elsewhere in the body.

^18^F-FDG uptake was also measured semiquantitatively as maximum standardized uptake value (SUV_max_) and target-to-background ratio (TBR). The SUV_max_ was measured in a volume of interest covering the CIED pocket area and in four positions via lead on co-registered CT images. The background radioactivity in the blood was measured from the ascending aorta excluding the vessel wall to calculate TBR. In addition to the visual and semiquantitative analysis we evaluated the ability to distinguish a CIED infection with a cut-off value of TBR ≥ 1.8 which was used in our previously published study in patients with a suspicion of prosthetic valve endocarditis.^15^

### Statistical Analysis

Continuous variables were characterized using means, standard deviations (SD) and range of values or medians and range of values for non-normally distributed variables, and in case of categorical variables frequencies and percentages were used. One-way ANOVA was used to test the differences between groups in continuous variables and Sidak´s method was used to adjust the *P* values of pairwise comparisons. For justification of the analyses, normality of the distributions were evaluated. *P* values less than .05 were considered as statistically significant. Statistical analyses were carried out using SAS system for Windows, Version 9.4 (SAS Institute Inc, Cary, NC, USA).

## Results

The mean age of patients with suspected CIED infection was 70 years (±13 years) and 23 (77%) were male. Background information of the patients is presented in Table 1 and time from CIED implantation or last cardiac device procedure to ^18^F-FDG-PET/CT in Table 2. Mean duration of antimicrobial treatment was 11 days (range 2-31 days, ± 7 days) before ^18^F-FDG-PET/CT. One patient didn’t receive antibiotics at the time of ^18^F-FDG-PET/CT.

Echocardiography was performed in all but seven patients who had only local symptoms (Table 2). 19 patients (63%) underwent transesophageal echocardiography and 5 (17%) transthoracic echocardiography. Vegetations or other findings indicating endocarditis were not seen on valves. Two patients had a mass raising suspicion of vegetation on the CIED lead. In follow-up the finding was considered to be thrombus in one case (Patient #17) and true bacterial vegetation in the other case (Patient # 18).

Background details of the control group are presented in Table 1 and Table 2. Mean age was 73 years (range 69-77 years, ± 3 years). These patients had neither suspicion of CIED or any other infection nor antibiotic treatment on PET/CT day.

### ^18^F-FDG-PET/CT in Control Group

In the control group, there were 10 patients who underwent ^18^F-FDG-PET/CT for evaluation of cancer. Visual analysis showed increased uptake of ^18^F-FDG associated with CIED in three cases: in the pocket area in two patients (SUV_max_ 3.4-3.5, TBR 1.4-1.8) and in the lead in one patient (SUV_max_ 3.5, TBR 1.4) (Table 2). By using the cut-off value of TBR ≥ 1.8 as a criterion for CIED infection, there were 9 true negative patients and one false positive patient. In the control group, mean SUV_max_ in the generator area was 2.0 ± .8 (TBR .9 ± .4) and in leads 2.4 ± .5 (TBR 1.0 ± .2). During follow-up (mean 3.7 years), none of the patients in control group presented with infectious symptoms of the CIED system.

### ^18^F-FDG-PET/CT in Suspected CIED Infection ≤ 8 Weeks After Implantation

In group 1, there were 9 patients with CIED implantation/intervention within ≤ 8 weeks and local signs of infection in the pocket area. Three of them had general signs of infection. In two, CIED pocket opened spontaneously. CIED was extracted in four patients in whom *Staphylococcus aureus* was identified as an etiological agent in blood culture (Patient #7 and #9) and/or in culture of the pocket site (Patient # 3, #7 and #8) indicating a definitive CIED infection. The other five cases were diagnosed with superficial skin infection that was treated with a short per oral antibiotic treatment. One of these patients (Patient #6) died soon after PET/CT due to sudden cardiac arrest and autopsy did not show signs of CIED infection. In the remaining 4 patients, signs of infection in the pocket area resolved and they didn’t show any signs of CIED infection during follow-up of 3 to 5 years.

By visual analysis, ^18^F-FDG-PET/CT was positive in all 9 cases. Six patients had uptake of ^18^F-FDG both in the pocket area and in leads, 2 patients only in the pocket area and 1 patient only in leads. Mean SUV_max_ and TBR at the generator pocket area as well as in leads were similar in patients with definitive CIED infection and patients with superficial infection (Table 3). By using the cut-off value of TBR ≥ 1.8 as a criterion for CIED infection, there were no false negative cases, but two false positive cases. Patients with a definitive CIED infection as well as patients with superficial infection had significantly higher SUV_max_ in the pocket area compared to the control group (*P* = <.0001, patients with definitive CIED infection and *P* = .010, patients with superficial infection). SUV_max_ and TBR values of the leads were similar to the control group (Table 3).

**Table 3:** Mean SUV and TBR values of the different groups and *P* values of comparison of patients with and without CIED infection and patients of control group

Group	SUV/TBR ±SD generator area	*P* value SUV/TBR	*P* value SUV/TBR study group vs control group	SUV/TBR ±SD Leads	*P* value SUV/TBR	*P* value SUV/TBR study group vs control group
Control group	2.0 ± .8/.9 ± .4			2.4 ± .5/1.0 ± .2		
Group 1 CIED infection	6.6 ± 1.0/2.8 ± .8	.103/.211	<.0001/.0002	4.9 ± 1.9/2.0 ± 1.0	.651/.507	.139/.232
Group 1 no CIED infection	4.4 ± 1.4/1.8 ± 1.0		.010/.121	3.1 ± .8/1.1 ± .3		.139/.232
Group 2 CIED infection	5.5 ± 2.2/2.3 ± 1.0	ND	<.0001/.0005	4.6 ± 3.2/2.9 ± 1.4	ND	.073/.069
Group 2 no CIED infection	2.3/1.5			1.5/1.0		
Group 3 CIED infection	2.3 ± .5/.8 ± .0	1.000/1.000	1.000/1.000	4.2 ± 1.4/1.7 ± .9	.958/.976	.774/.927
Group 3 no CIED infection	2.0 ± .5/.8 ± .2		1.000/.9999	3.0 ± 1.1/1.2 ± .4		.9897/.9999
Group 2 and Group 3 CIED infection	4.8 ± 2.4/2.0 ± 1.1	.0004/.005	.0005/.0090	4.6 ± 2.9/2.0 ± 1.3	.152/.103	.0476/.057
Group 2 and Group 3 no CIED infection	2.0 ± .4/.9 ± .3		1.0000/1.0000	2.8 ± 1.1/1.1 ± .4		.994/.9998

### ^18^F-FDG-PET/CT in Suspected CIED Infection > 8 Weeks After Implantation

In group 2, there were 9 patients with implantation/intervention of CIED > 8 weeks earlier (mean 4.6 years ± years, range 1.5-8 years) and clinical symptoms or signs of CIED infection; 7 with local signs of infection and 2 patients with possible endocarditis (Table 2). All of the patients with local signs had pain, swelling or erythema in CIED pocket area and 4 patients had a fistula at presentation. There were 8 patients with a definitive diagnosis of CIED infection and one patient with an alternative final diagnosis. CIED was removed from 7 patients all of whom were diagnosed as definitive CIED infection (bacterial cultures showed *Staphylococcus epidermidis* in 3 and *Corynebacterium* in one). In one patient with definitive CIED infection, device removal was withheld due to poor general condition, but *Staphylococcus aureus* was found in the purulent discharge from eroded pocket area. The patient without CIED infection showed a thrombotic mass in the pacemaker lead that was initially suspected as IE.

^18^F-FDG-PET/CT was positive in all 8 cases with a definitive CIED infection, but no uptake was detected in patient without CIED infection. In three cases, increased uptake of ^18^F-FDG was detected both in the generator area and leads, in four cases only in the generator area and in one case only in leads. The SUV_max_ and TBR in the pocket area were significantly higher in patients with a CIED infection than in the control group, but there was no difference in SUV_max_ and TBR in leads (Table 3). By using the cut-off value of TBR ≥ 1.8, there were 7 true positive and one false negative findings.

### ^18^F-FDG-PET/CT in FUO or Bacteremia

**Figure 1:**
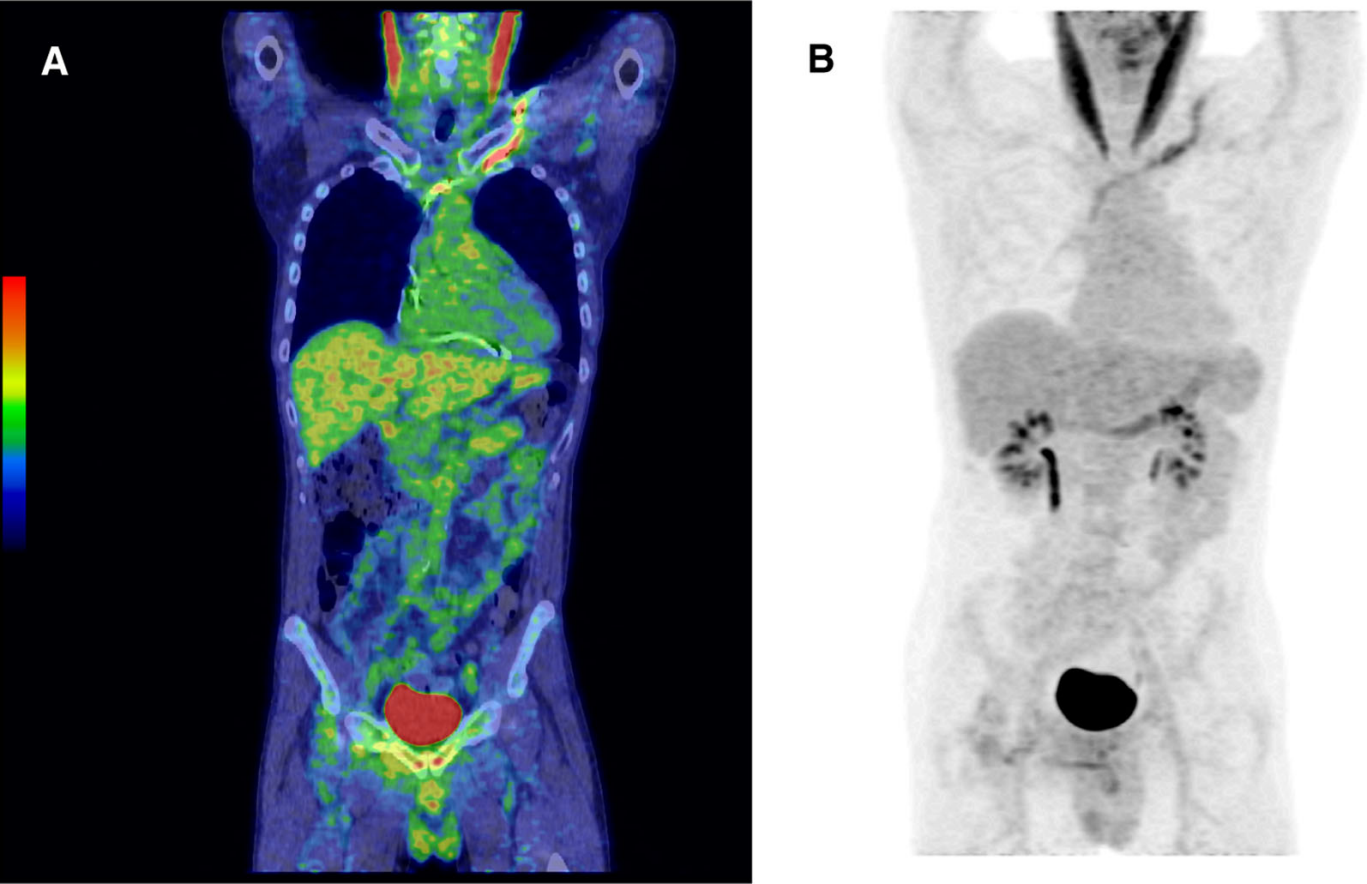
Patient #23 had fever of unknown origin, but no symptom in CIED area. The generator had been changed 1.5 years before. ^18^F-FDG-PET/CT showed uptake in lead, SUV_max_ 5.2 (Panel **A** and **B**). Bacterial culture from removed CIED system yielded *Staphylococcus epidermidis*.

Group 3 consisted of 12 patients without local signs of CIED infection, but FUO (N = 6) or bacteremia with typical endocardial pathogen or recurrent bacteremia with no identified focus (N = 6) (Table 2). Mean time from implantation of CIED was 2.4 years ± 2.6 years (range 2 months - 8 years). In 2 patients, definite CIED infection was diagnosed based on finding of *Staphylococcus epidermidis* in bacterial culture of the extracted generator. Ten cases were classified as having no CIED infection and they had uneventful follow-up (mean 3.7 years ± 1 year, range 2.5-5 years). There was increased ^18^F-FDG uptake in CIED leads in 4 patients, but only one of these had definitive CIED infection (Patient #23, Figure 1A, B). In one case (Patient #24), the lead uptake was associated with pericarditis. In 2 cases, *Staphylococcus aureus* bacteremia was treated with antibiotics successfully. In these 3 cases positive PET/CT finding of the lead was regarded as false positive. One of the 12 cases was regarded as false negative. In this case of FUO (Patient #22) *Staphylococcus epidermidis* was found in bacterial culture of the extracted system and PET/CT revealed hot spots in the lungs, which possibly presented infectious embolic foci in consequence to CIED endocarditis (Table 2).

In group 3, no difference was found in the mean SUV_max_ or TBR in 2 cases with definitive CIED infection compared to 10 patients with no CIED infection (Table 3). Furthermore, there was no difference between patients with definite CIED infection and the control group (Table 3). By using the cut-off value of TBR ≥ 1.8, there were no false positive cases, two true positive cases and one false negative case.

### Diagnostic Performance of ^18^F-FDG-PET/CT

**Figure 2:**
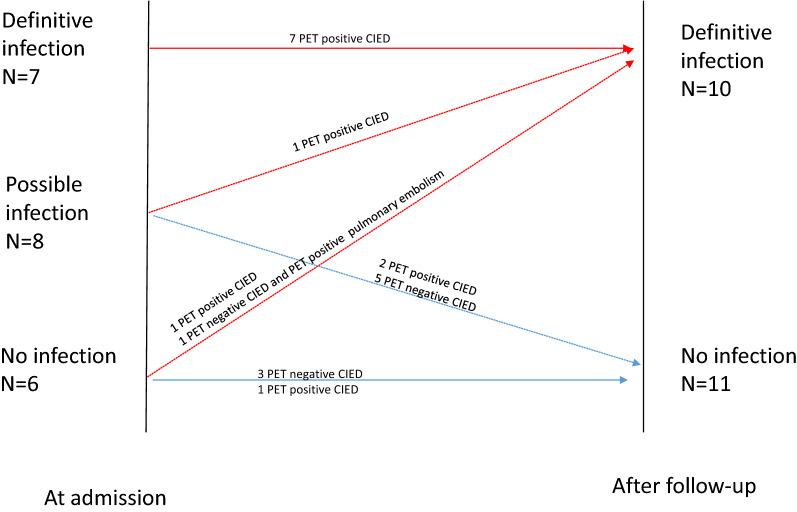
CIED infection case classification at admission and at the end of the follow-up of patients with suspicion of CIED infection and implantation/intervention of CIED > 8 weeks earlier (group 2 and group 3)

In patients with CIED implantation > 8 weeks and clinical symptoms or signs of CIED infection or FUO/bacteremia (groups 2 and 3), there was a significantly higher ^18^F-FDG uptake in the pocket area in patients with a definitive CIED infection than patients without CIED infection (SUV_max_ 4.8 ± 2.4 vs 2.0 ± .4, *P* = .0004 and TBR 2.0 ± 1.1 vs .9 ± .3, *P* = .005) or patients in the control group (*P* = .0005 and *P* = .009, respectively). However, there were no differences in ^18^F-FDG uptake in leads in patients with CIED infection compared to patients without CIED infection (Table 3). SUV_max_ value of leads was higher in patients with CIED infection compared to control group (SUV_max_ 4.6 ± 2.9 vs 2.4 ± .5, *P* = .048) but there was no significant difference in TBR value (Table 3). In groups 2, 3, the cut-off value of TBR ≥ 1.8 (either in the pocket area or leads) resulted in sensitivity of 90%, specificity of 73%, positive predictive value (PPV) of 75%, and negative predictive value (NPV) of 89% for the detection of definitive CIED infection. As Figure 2 shows, ^18^F-FDG-PET/CT enabled to correctly reclassify and achieve a conclusive diagnosis in 6 of the 8 patients initially classified as possible CIED infection. In addition the result of ^18^F-FDG-PET/CT correctly reclassified one patient with no CIED infection at admission and definitive infection at the end of the follow-up.

### Extracardiac Findings in ^18^F-FDG-PET/CT

Six of the 14 patients with definite CIED infection had active lymph nodes in mediastinum or axillary region indicating active infection in thoracic region and 2 patients with CIED infection had uptake of ^18^F-FDG in lungs. Patient #26 had also uptake of ^18^F-FDG in descending colon and this finding was confirmed as a tubular adenoma in colonoscopy and biopsies later on. PET/CT revealed other causes of FUO and infectious foci in patients with bacteremia: pericarditis in Patient #24, pneumonia in Patient #20, urinary retention and epididymitis in Patient #21, arthritis and small intramuscular abscesses in Patient #25, osteomyelitis in Patient #26, and intramuscular abscess in Patient #29.

## Discussion

Multimodality imaging may help in the diagnostics of endocarditis, but the value of ^18^F-FDG-PET/CT in CIED infection still remains uncertain.^5^ The lack of gold standard for defining CIED infection poses a challenge for the evaluation of a new diagnostic method. Our study adds to the previous studies on the value of ^18^F-FDG-PET/CT in patients with suspicion of CIED infection (18-20) in showing that it has high sensitivity and moderate specificity in the presence of suspected CIED infection > 8 weeks after device implantation.

To avoid unnecessary device removal in patients with recent implantation of CIED the most important thing is to differentiate whether the patient has superficial or deeper pocket infection. Unfortunately, in our study, ^18^F-FDG-PET/CT was of limited value in this respect. Patients in group 1 had recently implanted CIED and all had hot spots either in pocket area or in leads and no significant difference was found in SUV_max_ values between patients with CIED infection and superficial infection. Somewhat better differentiation was achieved using the cut-off value of TBR ≥ 1.8 with two false positive cases compared to the visual analysis with 5 false positive cases. According to our results positive uptake of ^18^F-FDG found in PET/CT within 8 weeks after implantation of CIED should be interpreted with caution. Uptake of ^18^F-FDG can occur also due to inflammation and normal wound healing process after implantation. Recent meta-analysis also pointed out the difficulties of interpreting ^18^F-FDG-PET/CT findings as infection or inflammation after recent implantation.^16^

**Figure 3:**
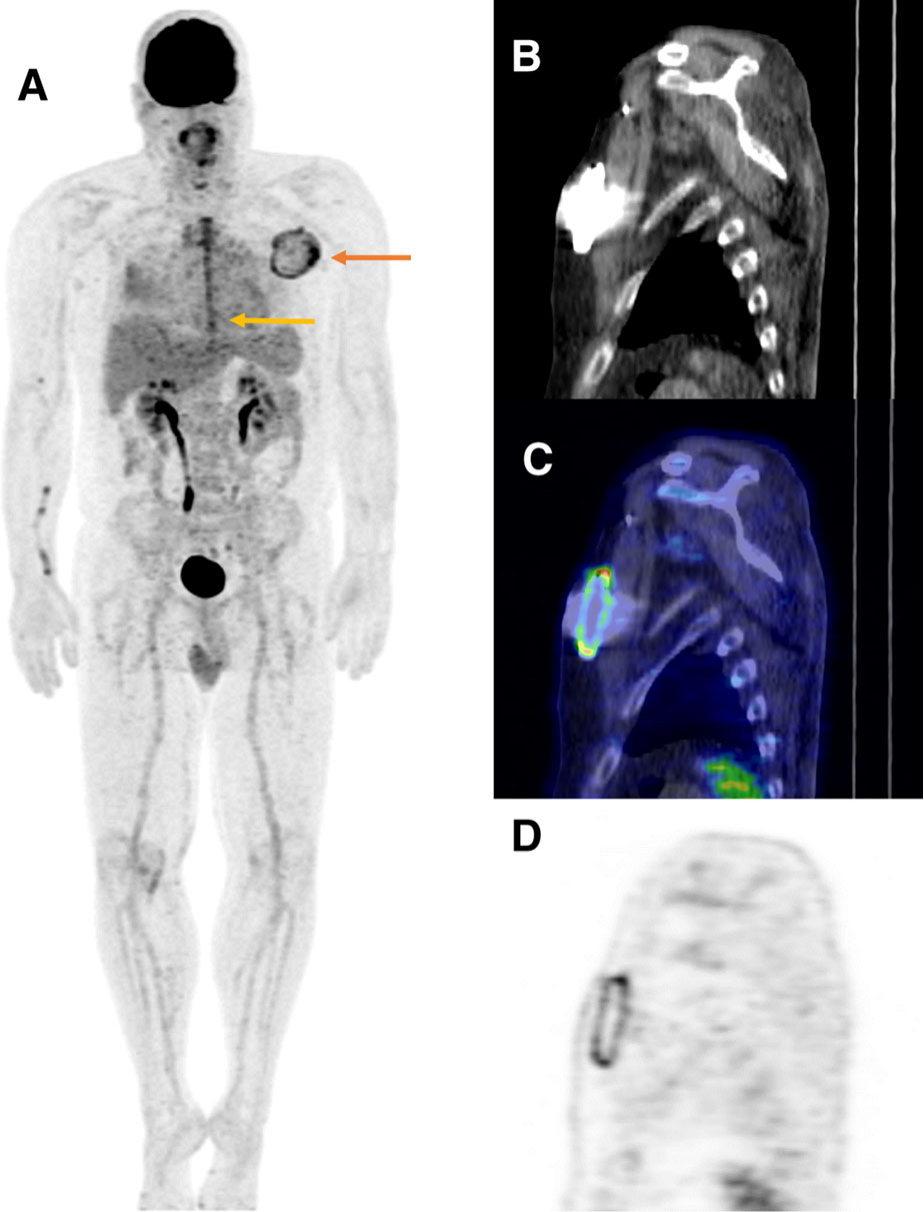
Patient #10 had CIED implanted 8 years ago and now two months after elective CABG procedure he had pain, redness and swelling in pocket area. ^18^F-FDG-PET/CT showed uptake in pocket (SUV_max_ 7.1) (Panel **A**, *red arrow*). There were also physiological uptake in sternotomy wound (SUV_max_ 5.3) (Panel A, *yellow arrow*). The CIED system was removed and infection was confirmed as bacterial culture which yielded *Staphylococcus epidermidis.* Panel **B** sagittal CT scan, Panel **C** sagittal fusion, Panel **D** sagittal PET

^18^FDG-PET/CT was accurate in the detection of CIED infection in patients who had device implanted > 8 weeks earlier. ^18^FDG-PET/CT was positive in all 8 cases with definitive CIED infection in group 2. In addition, the SUV_max_ and TBR values were significantly higher compared to the control group with no infection. Patient #10 for example had high ^18^F-FDG uptake (SUV_max_ 7.1) in pocket area and bacterial culture confirmed CIED infection (Figure 3). In this group only, the cut-off value of TBR ≥ 1.8 was 88% sensitive and 90% specific. It is of note that this group included two patients who fulfilled the diagnostic criteria of infective endocarditis by traditional methods and ^18^FDG-PET/CT correctly confirmed it in the other case and excluded in other.

Fever of unknown origin remains a challenge to clinicians. In a French study of FUO patients with CIED, most patients had ^18^F-FDG uptake in the CIED system indicating an ongoing infection that was confirmed by microbiological samples taken at the time of CIED extraction.^17^ This type of silent infection appears in all kinds of foreign materials. In the present study, ^18^F-FDG-PET/CT revealed a significant CIED related or other diagnostic finding in every patient in the FUO patients of group 3 (see above, Results: Other findings and Table 2). Concerning CIED infections among this group there was one true positive, one false positive and one false negative PET/CT. In the false positive case, pericarditis causing accumulation of ^18^F-FDG in the heart was diagnosed. In the false negative case, patient with a definitive CIED infection caused by *Staphylococcus epidermidis* had received antibiotics for 11 days before PET/CT, which could have explained the absence of ^18^F-FDG uptake. In two cases with CIED infection ^18^F-FDG-PET/CT revealed hotspots in lungs indicating embolic foci of CIED infection. The usefulness of ^18^F-FDG-PET/CT to detect septic embolisms or metastatic infectious foci in CIED endocarditis was shown in a previous study.^18^ We had 6 patients with bacteremia with no identified focus. In 2 cases with *Staphylococcus aureus* bacteremia, uptake of ^18^F-FDG was detected in leads besides other infectious foci detected by PET/CT. CIED systems were not extracted and patients got long antibiotic treatment. Although we are not able to exclude the possibility CIED infection and successful treatment of CIED infection conservatively with antibiotics, these cases were not diagnosed with definitive CIED infection due to uneventful clinical course and the presence of other obvious infectious foci in PET/CT. PET/CT revealed infectious foci also in three additional patients with bacteremia. As shown in our study and previous studies,^7^,^10^,^11^,^17^ in the group of CIED patients with FUO or bacteremia without identified focus, ^18^F-FDG-PET/CT may help to identify or exclude CIED infection and reveal other infectious foci, inflammatory diseases and malignancy. Clinically important advantage is the high negative predictive value. There was only one false negative case among the 12 patients with FUO or bacteremia. Ours as well as previous results suggest that in patients with a suspicion of CIED infection and negative finding of ^18^FDG-PET/CT, the extraction may be withheld with close monitoring during and after antimicrobial treatment.^10^

^18^F-FDG-PET/CT has limitations when imaging CIED infections. The specificity of ^18^F-FDG-PET/CT to distinguish infection from inflammation is low which is also reflected by lower specificity in patients with CIED implantation < 8 weeks earlier.^10^,^19^ Also in the control group, there were three cases with mild uptake of ^18^F-FDG in the pocket area or leads (one with TBR ≥ 1.8). All these were false positives as there were no signs of infection neither before nor after PET/CT. The false positive signal may be related to reactive adjacent lymph nodes that are difficult to differentiate anatomically. Pacemaker leads are thin objects and thus leukocyte accumulation and ^18^F-FDG uptake around leads can be mild leading to false negative findings.^6^-^8^^18^F-FDG uptake can also be diminished if a patient has received antibiotic treatment before PET/CT. In this study, all except one patient had antibiotic treatment ongoing at the time of PET/CT and mean time from starting it was 11 days (range 2-31 days). The problem of previous antibiotic treatment has also been a concern in previous studies.^7^,^9^

In the present study, the patient population was small and clinical presentation varied. On the other hand these patients represent real life with the challenges that clinicians meet. Another limitation is the limited number of microbiological cultures of the extracted CIED systems. Also microbiological samples remained negative even in some clinically definitive CIED infections. As in clinical practice some of the diagnosis end exclusions of CIED infection were based on clinical judgment. However, the follow-up time of our study was long enough to find out false negative judgments of CIED infection. The criteria for positivity of FDG-PET imaging was defined and validated retrospectively in the same group of patients. As a result no firm conclusions can be driven based on our results and cut-off value of TBR should be tested prospectively in another patient cohort.

## Conclusions

^18^FDG-PET/CT demonstrates high sensitivity and moderate specificity for the detection of CIED infection >8 weeks after device implantation. However, in patients with recent implantation or other intervention of CIED, the accumulation of ^18^F-FDG in CIED must be interpreted with caution due to low specificity. In patients with FUO or bacteremia, ^18^F-FDG-PET/CT may be helpful in identification of CIED infection as well as alternative diagnoses.

## New Knowledge Gained

Among patients with recent (≤ 8weeks) implantation or intervention of CIED system ^18^FDG-PET/CT can’t differentiate superficial and deep infection. ^18^FDG-PET/CT is highly sensitive for the detection of CIED infection and has high negative predictive value to rule out CIED infection > 8 weeks after implantation/intervention of CIED.

## Electronic supplementary material

Below is the link to the electronic supplementary material.

Supplementary material 1 (PPTX 914 kb)

Supplementary material 2 (M4A 4920 kb)

## Abbreviations

^18^F-FDG: 2-[^18^F]fluoro-2-deoxy-*D*-glucose
PET/CT: Positron emission tomography/computed tomography
CIED: Cardiac implantable electronic device
SUV_max_: Standardized uptake value
TBR: Target-to-background ratio

## References

[1] Sohail MR, Henrikson CA, Braid-Forbes MJ, Forbes KF, Lerner DJ (2011). Mortality and cost associated with cardiovascular implantable electronic device infections. Arch Intern Med..

[2] Tarakji KG, Chan EJ, Cantillon DJ, Doonan AL, Hu T, Schmitt S (2010). Cardiac implantable electronic device infections: Presentation, management, and patient outcomes. HeartRhythm.

[3] Wilkoff BL, Love CJ, Byrd CL, Bongiorni MG, Carrillo RG, Crossley GH (2009). Transvenous lead extraction: Heart Rhythm Society expert consensus on facilities, training, indications, and patient management: This document was endorsed by the American Heart Association (AHA). Heart Rhythm.

[4] Baddour LM, Epstein AE, Erickson CC, Knight BP, Levison ME, Lockhart PB (2010). Update on cardiovascular implantable electronic device infections and their management: A scientific statement from the American Heart Association. Circulation.

[5] Habib G, Lancellotti P, Antunes MJ, Bongiorni MG, Casalta JP, Del-Zotti F (2015). 2015 ESC Guidelines for the management of infective endocarditis: The Task Force for the Management of Infective Endocarditis of the European Society of Cardiology (ESC). Endorsed by: European Association for Cardio-Thoracic Surgery (EACTS), the European Association of Nuclear Medicine (EANM). Eur Heart J.

[6] Leccisotti L, Perna F, Lago M, Leo M, Stafanelli A, Calcagni ML (2014). Cardiovascular implantable electronic device infection: Delayed vs standard FDG PET-CT imaging. J Nucl Cardiol.

[7] Bensimhon L, Lavergne T, Hugonnet F, Mainardi JL, Latremouille C, Maunoury C (2011). Whole body [(18)F]fluorodeoxyglucose positron emission tomography imaging for the diagnosis of pacemaker or implantable cardioverter defibrillator infection: A preliminary prospective study. Clin Microbiol Infect..

[8] Cautela J, Alessandrini S, Cammilleri S, Giorgi R, Richet H, Casalta JP (2013). Diagnostic yield of FDG positron-emission tomography/computed tomography in patients with CEID infection: A pilot study. Europace.

[9] Ahmed FZ, James J, Cunnington C, Motwani M, Fullwood C, Hooper J (2015). Early diagnosis of cardiac implantable electronic device generator pocket infection using ^18^F-FDG-PET/CT. Eur Heart J Cardiovasc Imaging..

[10] Sarrazin JF, Philippon F, Tessier M, Guimond J, Molin F, Champagne J (2012). Usefulness of Fluorine-18 Positron Emission Tomography/Computed Tomography for Identification of Cardiovascular Implantable Electronic Device Infections. J Am Coll Cardiol..

[11] Tlili G, Amraoui S, Mesguich C, Rivière A, Bordachar P, Hindié E, Bordenave L (2015). High performances of (18)F-fluorodeoxyglucose PET-CT in cardiac implantable device infections: A study of 40 patients. J Nucl Cardiol..

[12] Li JS, Sexton DJ, Mick N, Nettles R, Fowler VG, Ryan T (2000). Proposed modifications to the Duke criteria for the diagnosis of infective endocarditis. Clin Infect Dis.

[13] Williams G, Kolodny GM (2008). Suppression of myocardial 18F-FDG uptake by preparing patients with a high-fat, low-carbohydrate diet. AJR Am J Roentgenol.

[14] Osborne MT, Hulten EA, Murthy VL, Skali H (2017). Patient preparation for cardiac fluorine-18 fluorodeoxyglucose positron emission tomography imaging of inflammation. J Nucl Cardiol..

[15] Salomäki SP, Saraste A, Kemppainen J, Bax JJ, Knuuti J, Nuutila P (2017). ^18^F-FDG positron emission tomography/computed tomography in infective endocarditis. J Nucl Cardiol.

[16] Mahmood M, Kendi AT, Farid S, Ajmal S (2019). Role of ^18^F-FDG PET/CT in the diagnosis of cardiovascular implantable electronic device infections: A meta-analysis. J Nucl Cardiol..

[17] Ploux S, Riviere A, Amraoui S, Whinnett Z, Barandon L, Lafitte S (2011). Positron emission tomography in patients with suspected pacing system infections may play a critical role in difficult cases. Heart Rhythm.

[18] Amraoui S, Tlili G, Sohal M, Berte B, Hindié E, Ritter P (2016). Contribution of PET imaging to the diagnosis of septic embolism in patients with pacing lead endocarditis. JACC Cardiovasc Imaging..

[19] Vaidyanathan S, Patel CN, Scarsbrook AF, Chowdhury FU (2015). FDG PET/CT in infection and inflammation—current and emerging clinical applications. Clin Radiol..

