# Supplementary material for: 18F-FDG positron emission tomography/computed tomography of cardiac implantable electronic device infections
Source: J Nucl Cardiol. 2020 Jul 31;28(6):2992–3003. doi: 10.1007/s12350-020-02256-4 (PMC8709812; doi:10.1007/s12350-020-02256-4)
Supplement: Supplementary file 1 — Supplementary material 1 (PPTX 914 kb) [file 12350_2020_2256_MOESM1_ESM.pptx]

## Slide 1
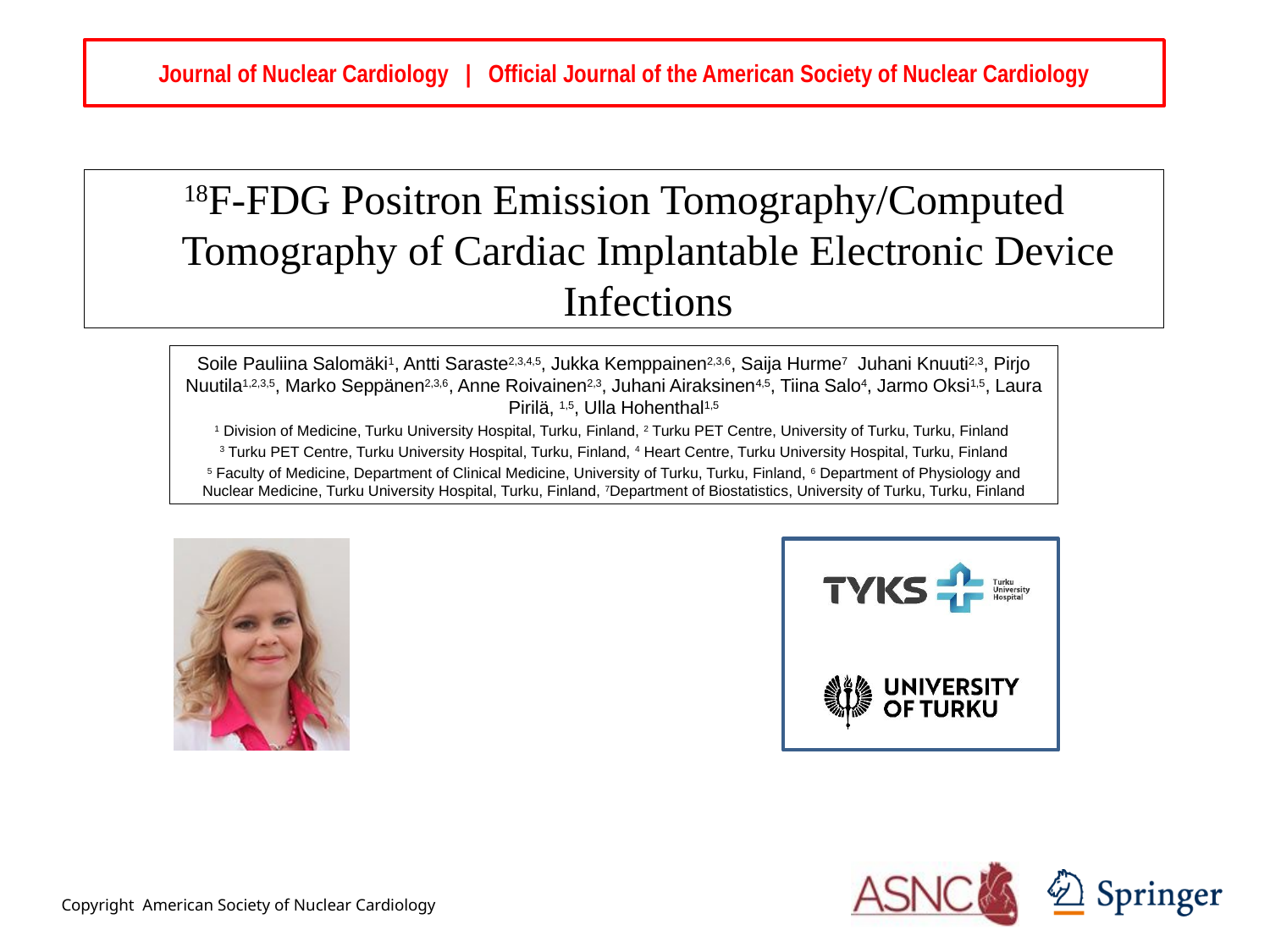

Journal of Nuclear Cardiology | Official Journal of the American Society of Nuclear Cardiology
# 18F-FDG Positron Emission Tomography/Computed Tomography of Cardiac Implantable Electronic Device Infections
Soile Pauliina Salomäki1, Antti Saraste2,3,4,5, Jukka Kemppainen2,3,6, Saija Hurme7 Juhani Knuuti2,3, Pirjo Nuutila1,2,3,5, Marko Seppänen2,3,6, Anne Roivainen2,3, Juhani Airaksinen4,5, Tiina Salo4, Jarmo Oksi1,5, Laura Pirilä, 1,5, Ulla Hohenthal1,5
1 Division of Medicine, Turku University Hospital, Turku, Finland, 2 Turku PET Centre, University of Turku, Turku, Finland
3 Turku PET Centre, Turku University Hospital, Turku, Finland, 4 Heart Centre, Turku University Hospital, Turku, Finland
5 Faculty of Medicine, Department of Clinical Medicine, University of Turku, Turku, Finland, 6 Department of Physiology and Nuclear Medicine, Turku University Hospital, Turku, Finland, 7Department of Biostatistics, University of Turku, Turku, Finland
Copyright American Society of Nuclear Cardiology

## Slide 2
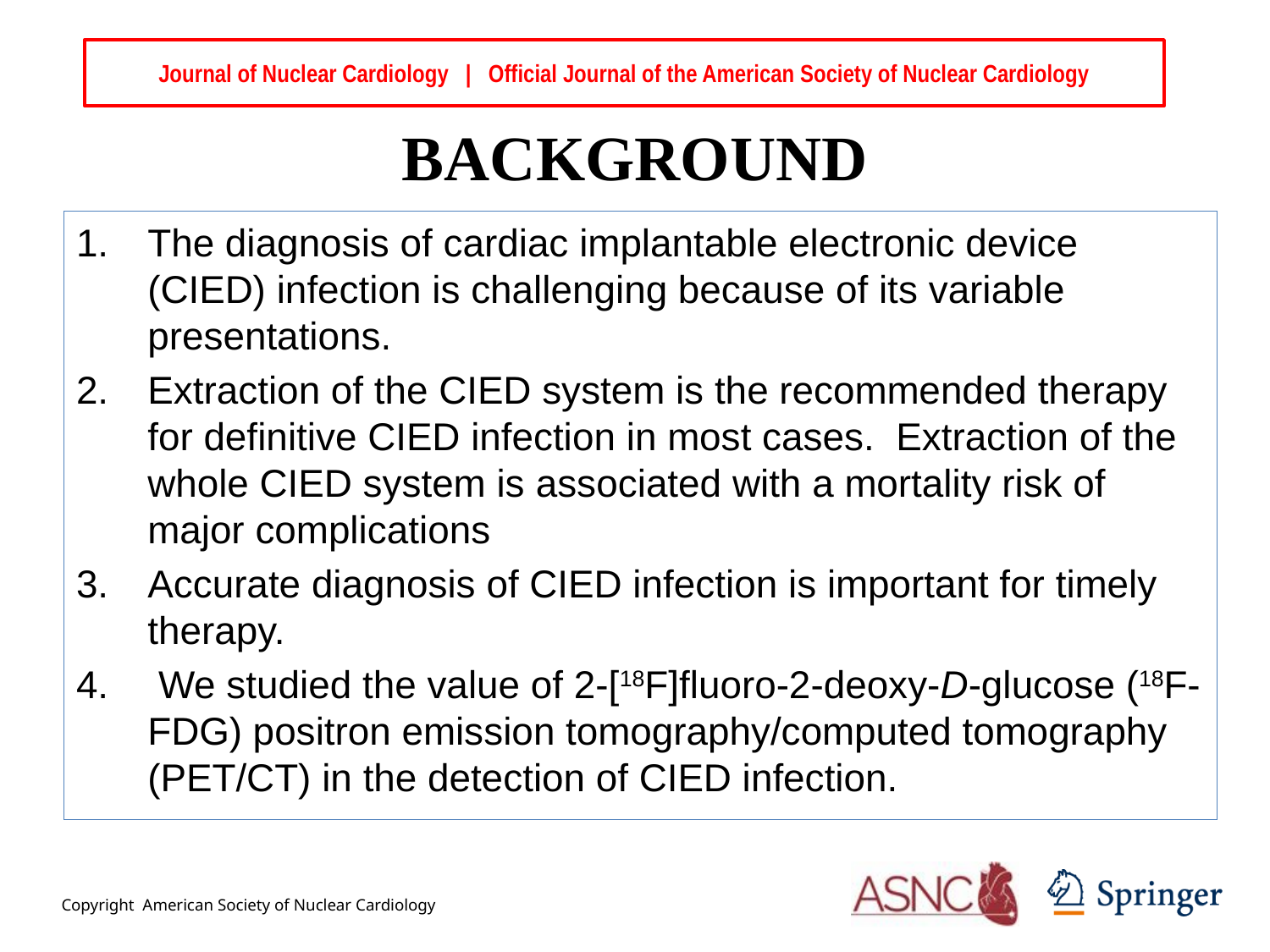

Journal of Nuclear Cardiology | Official Journal of the American Society of Nuclear Cardiology
# BACKGROUND
The diagnosis of cardiac implantable electronic device (CIED) infection is challenging because of its variable presentations.
Extraction of the CIED system is the recommended therapy for definitive CIED infection in most cases. Extraction of the whole CIED system is associated with a mortality risk of major complications
Accurate diagnosis of CIED infection is important for timely therapy.
 We studied the value of 2-[18F]fluoro-2-deoxy-D-glucose (18F-FDG) positron emission tomography/computed tomography (PET/CT) in the detection of CIED infection.
Copyright American Society of Nuclear Cardiology

## Slide 3
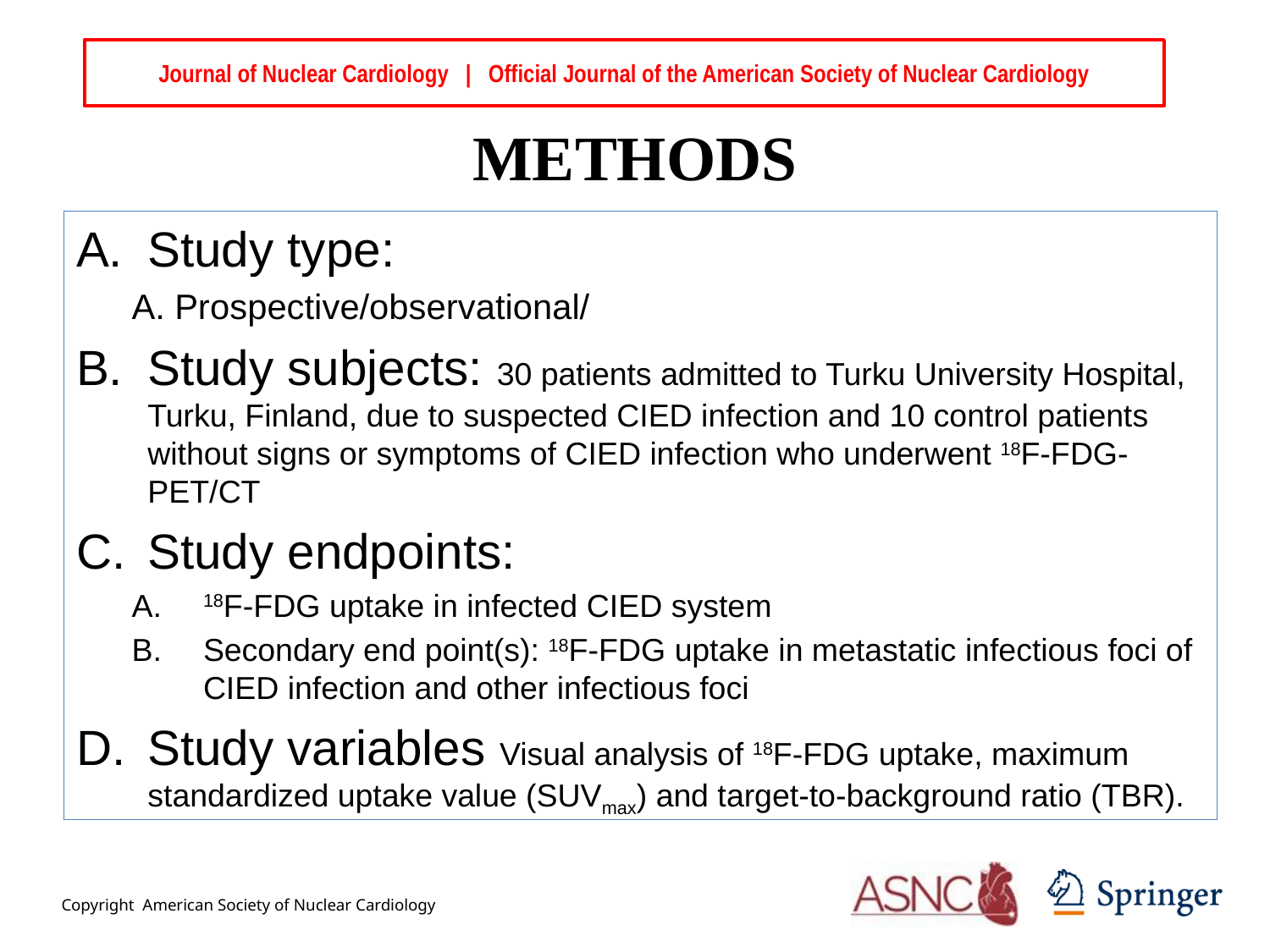

Journal of Nuclear Cardiology | Official Journal of the American Society of Nuclear Cardiology
# METHODS
Study type:
A. Prospective/observational/
Study subjects: 30 patients admitted to Turku University Hospital, Turku, Finland, due to suspected CIED infection and 10 control patients without signs or symptoms of CIED infection who underwent 18F-FDG-PET/CT
Study endpoints:
18F-FDG uptake in infected CIED system
Secondary end point(s): 18F-FDG uptake in metastatic infectious foci of CIED infection and other infectious foci
Study variables Visual analysis of 18F-FDG uptake, maximum standardized uptake value (SUVmax) and target-to-background ratio (TBR).
Copyright American Society of Nuclear Cardiology

## Slide 4
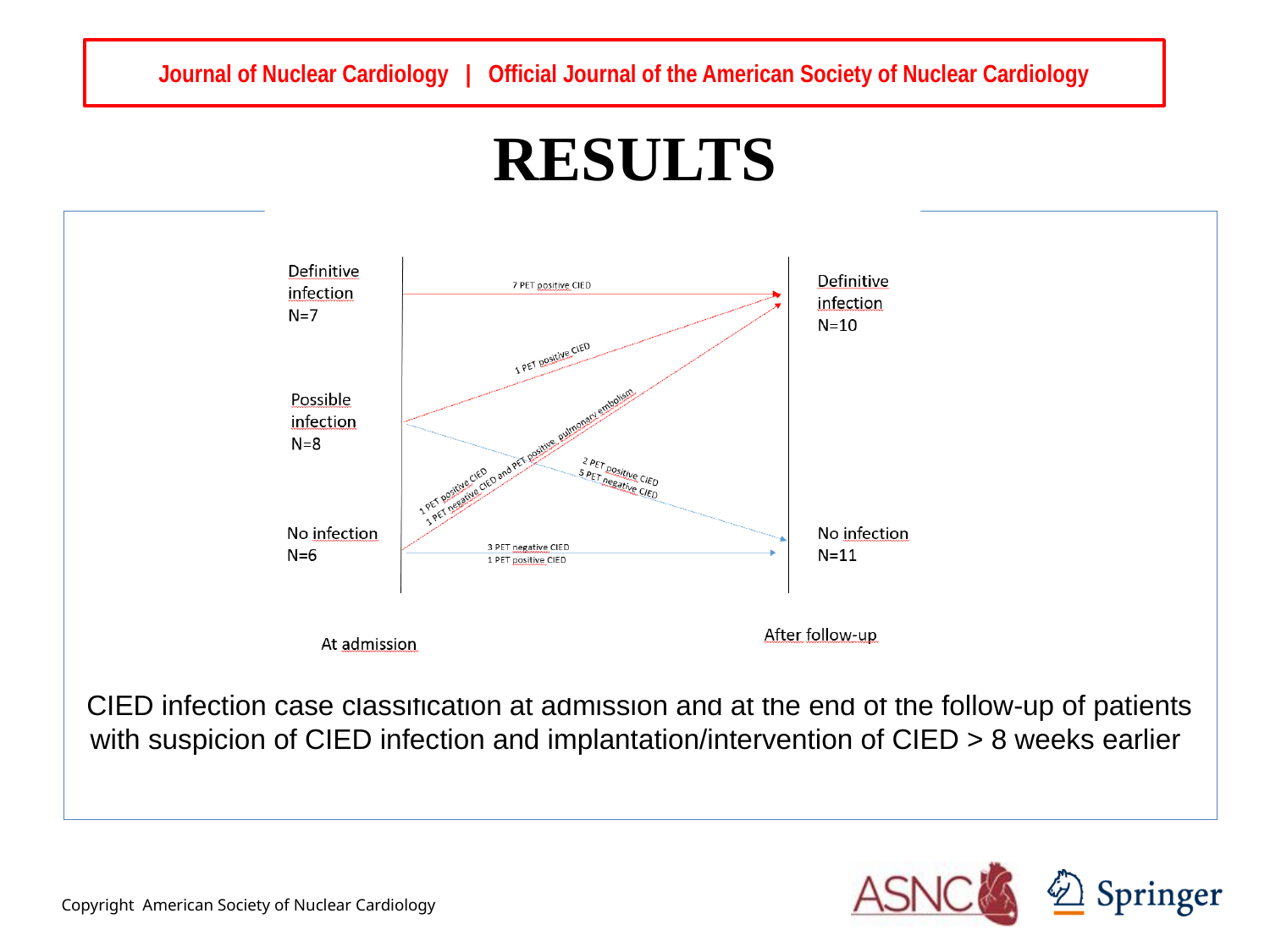

Journal of Nuclear Cardiology | Official Journal of the American Society of Nuclear Cardiology
# RESULTS
If figure, insert legend
CIED infection case classification at admission and at the end of the follow-up of patients with suspicion of CIED infection and implantation/intervention of CIED > 8 weeks earlier
Copyright American Society of Nuclear Cardiology

## Slide 5
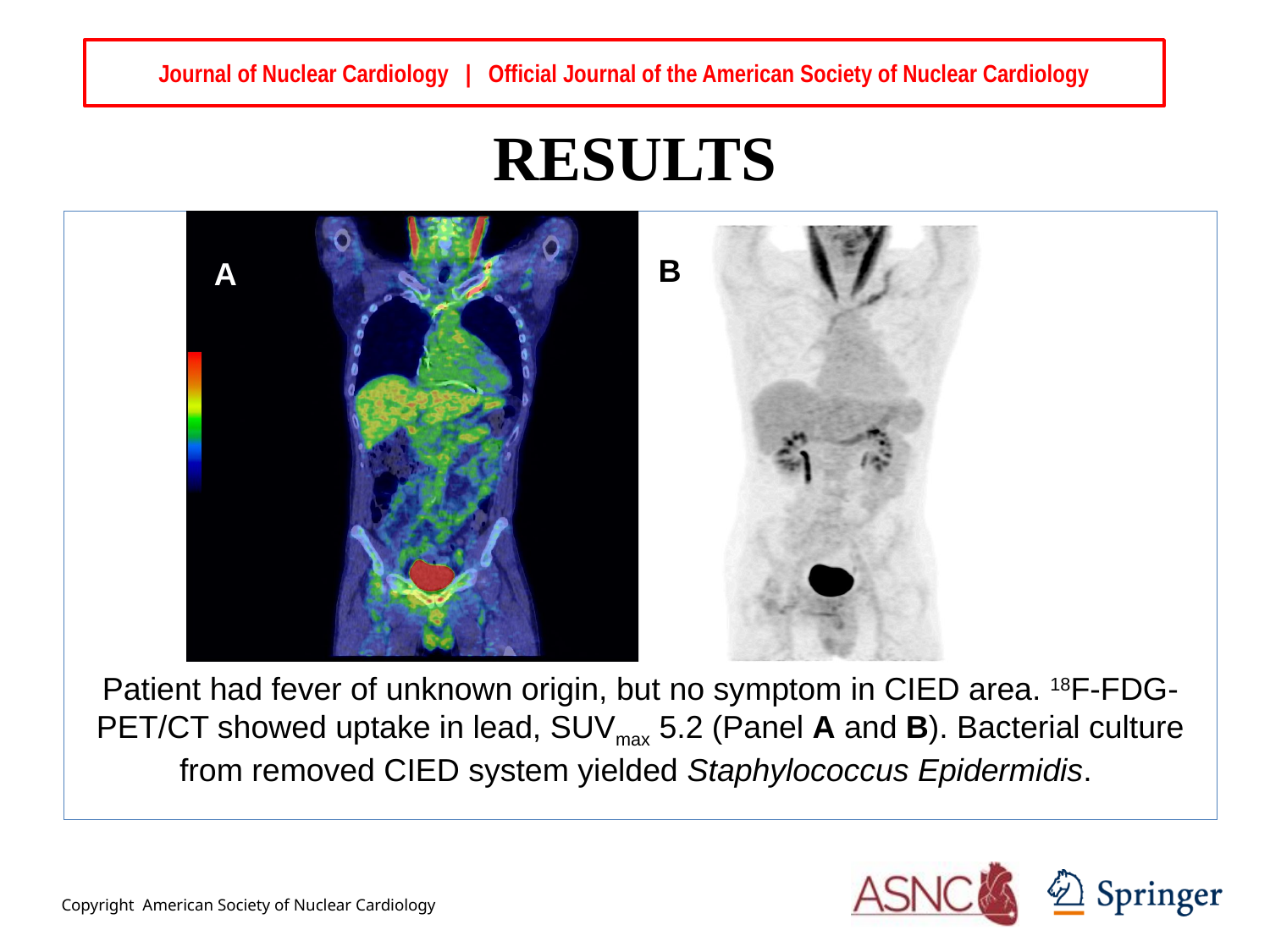

Journal of Nuclear Cardiology | Official Journal of the American Society of Nuclear Cardiology
# RESULTS
Patient had fever of unknown origin, but no symptom in CIED area. 18F-FDG-PET/CT showed uptake in lead, SUVmax 5.2 (Panel A and B). Bacterial culture from removed CIED system yielded Staphylococcus Epidermidis.
B
A
Copyright American Society of Nuclear Cardiology

## Slide 6
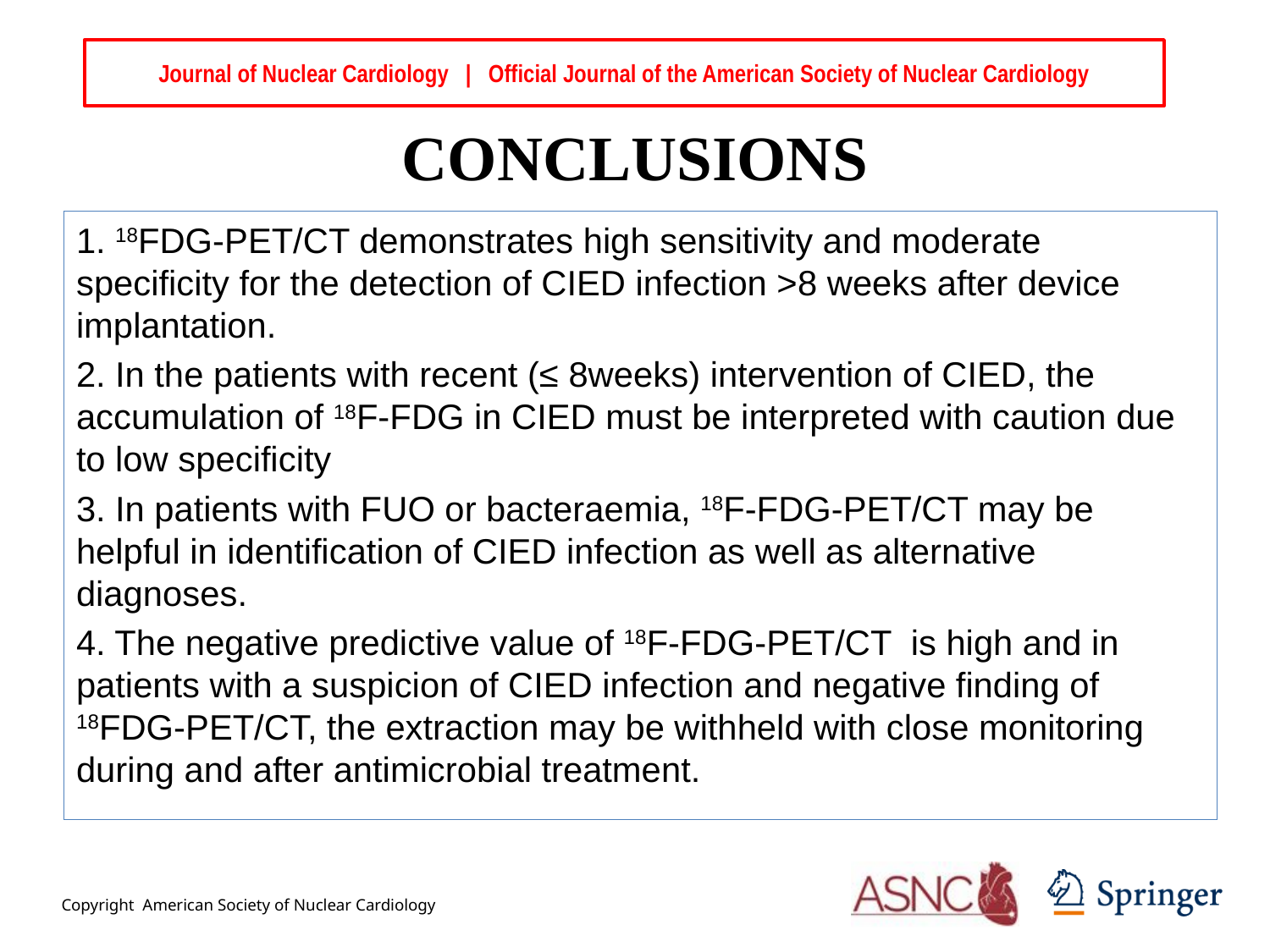

Journal of Nuclear Cardiology | Official Journal of the American Society of Nuclear Cardiology
# CONCLUSIONS
1. 18FDG-PET/CT demonstrates high sensitivity and moderate specificity for the detection of CIED infection >8 weeks after device implantation.
2. In the patients with recent (≤ 8weeks) intervention of CIED, the accumulation of 18F-FDG in CIED must be interpreted with caution due to low specificity
3. In patients with FUO or bacteraemia, 18F-FDG-PET/CT may be helpful in identification of CIED infection as well as alternative diagnoses.
4. The negative predictive value of 18F-FDG-PET/CT is high and in patients with a suspicion of CIED infection and negative finding of 18FDG-PET/CT, the extraction may be withheld with close monitoring during and after antimicrobial treatment.
Copyright American Society of Nuclear Cardiology
